# Potential drug targets for asthma identified in the plasma and brain through Mendelian randomization analysis

**DOI:** 10.3389/fimmu.2023.1240517

**Published:** 2023-09-21

**Authors:** Yuting Wang, Jiaxi Wang, Zhanfeng Yan, Siming Liu, Wenlong Xu

**Affiliations:** ^1^ Department of Otorhinolaryngology, Dongfang Hospital Affiliated to Beijing University of Chinese Medicine, Beijing, China; ^2^ Department of Otorhinolaryngology, Dongzhimen Hospital Affiliated to Beijing University of Chinese Medicine, Beijing, China

**Keywords:** asthma, Mendelian randomization, drug target, neurogenic inflammation, protein quantitative trait loci

## Abstract

**Background:**

Asthma is a heterogeneous disease, and the involvement of neurogenic inflammation is crucial in its development. The standardized treatments focus on alleviating symptoms. Despite the availability of medications for asthma, they have proven to be inadequate in controlling relapses and halting the progression of the disease. Therefore, there is a need for novel drug targets to prevent asthma.

**Methods:**

We utilized Mendelian randomization to investigate potential drug targets for asthma. We analyzed summary statistics from the UK Biobank and then replicated our findings in GWAS data by Demenais et al. and the FinnGen cohort. We obtained genetic instruments for 734 plasma and 73 brain proteins from recently reported GWAS. Next, we utilized reverse causal relationship analysis, Bayesian co-localization, and phenotype scanning as part of our sensitivity analysis. Furthermore, we performed a comparison and protein–protein interaction analysis to identify causal proteins. We also analyzed the possible consequences of our discoveries by the given existing asthma drugs and their targets.

**Results:**

Using Mendelian randomization analysis, we identified five protein–asthma pairs that were significant at the Bonferroni level (*P* < 6.35 × 10^−5^). Specifically, in plasma, we found that an increase of one standard deviation in IL1R1 and ECM1 was associated with an increased risk of asthma, while an increase in ADAM19 was found to be protective. The corresponding odds ratios were 1.03 (95% CI, 1.02–1.04), 1.00 (95% CI, 1.00–1.01), and 0.99 (95% CI, 0.98–0.99), respectively. In the brain, per 10-fold increase in ECM1 (OR, 1.05; 95% CI, 1.03–1.08) and PDLIM4 (OR, 1.05; 95% CI, 1.04–1.07) increased the risk of asthma. Bayesian co-localization found that ECM1 in the plasma (coloc.abf-PPH4 = 0.965) and in the brain (coloc.abf-PPH4 = 0.931) shared the same mutation with asthma. The target proteins of current asthma medications were found to interact with IL1R1. IL1R1 and PDLIM4 were validated in two replication cohorts.

**Conclusion:**

Our integrative analysis revealed that asthma risk is causally affected by the levels of IL1R1, ECM1, and PDLIM4. The results suggest that these three proteins have the potential to be used as drug targets for asthma, and further investigation through clinical trials is needed.

## 1 Introduction

Asthma is a widespread chronic disease that affects 358 million people globally (1). Severe asthma is a condition that affects 5% to 10% of the global asthma population. Unfortunately, approximately 3.6% of asthma patients still experience poor asthma control, even with standardized treatment (2). Additionally, patients with severe asthma have higher proportions of anxiety, depression, and hyperventilation syndrome. This can lead to patients feeling overwhelmed, with 8% of severe asthma patients attempting suicide (3).

On the other hand, stress, anxiety, and depression can exacerbate asthma. This may be the result of changes in the central or peripheral nervous system (such as the insula cortex and the HPA axis) in reaction to stress and mental illness. Thus, the neuroendocrine system produces stress hormones that can exacerbate asthma (4). Asthma is a complex inflammatory disease of the airways that is heterogeneous due to numerous natural and social environmental factors as well as an individual’s genetic makeup (5).

Treatment options for people with severe asthma are limited and have adverse side effects. Current research is mainly focused on personalized targeted therapy involving molecular biological agents such as genes and proteins. This approach offers hope to patients. Biological therapies target specific inflammatory pathways involved in the development of asthma, especially in patients with type 2 (T2) inflammation (6). Now, there are over 12 modifying treatments available that target the inflammatory component of the disease, including anti-IgE monoclonal antibody, anti-interleukin monoclonal antibodies, anti-thymic stromal lymphopoietin (TSLP) monoclonal antibodies, prostaglandin D2 receptor 2 (PTGDR2) selective competitive antagonists, and GATA binding protein 3 (GATA3)-specific DNAzyme (7, 8). However, the treatment of neuroimmune-related asthma exacerbation still lacks effective targets.

Mendelian randomization (MR) is a method of using genetic variation associated with exposures or risk factors to estimate a likely causal relationship to an outcome. This approach aims to minimize the impact of confounding factors, including reverse causation, in epidemiological studies (9). With the advancement of multi-omics research, the investigation of plasma and brain tissue proteomics has become increasingly extensive. Drug target research using MR has been widely applied in various diseases, including research that depends on expression quantitative trait locus (eQTL) and GWAS (10). Despite the influence of linkage disequilibrium (LD), local genetic interaction (cis-eQTL) still plays a significant role in regulating the expression of most genes (11). Variant protein quantitative trait loci (pQTLs) that affect protein levels are often located far away from coding genes, which partially overcomes the limitation of a few instruments (12). According to a previous study, the likelihood of a protein drug target receiving market approval was twice as high if it possessed genetic support and guided selection, compared with those without such support (13). Nevertheless, there are few studies on asthma drug targets based on GWAS and pQTL data and no report on the analysis of asthma drug targets based on pQTL in plasma and brain tissue.

**Figure 1:**
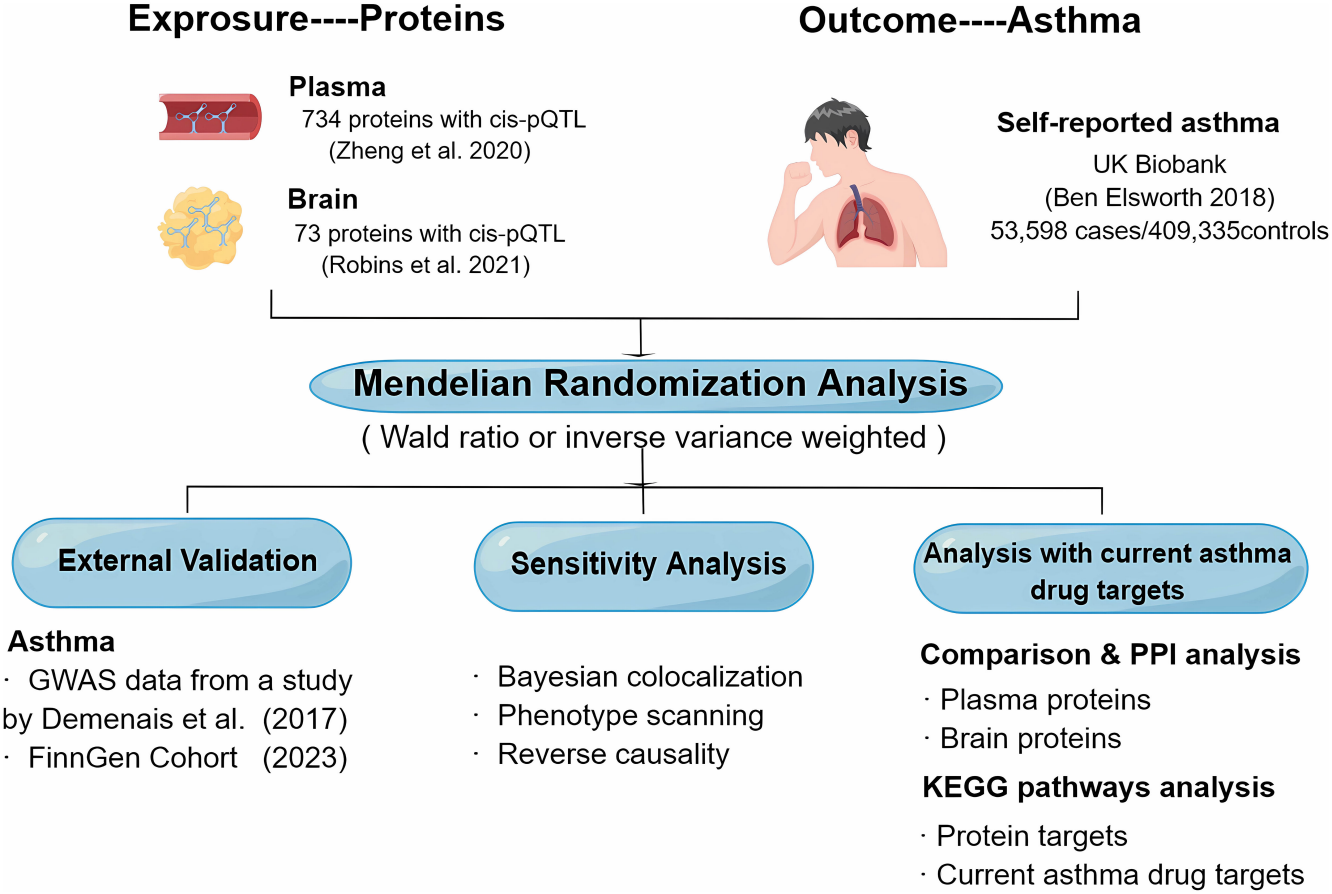
Study design for the identification of plasma and brain proteins causally associated with asthma.

The objective of this study was to investigate the potential therapeutic targets for asthma by identifying plasma together with brain proteins. We used MR to identify potential causal plasma and brain proteins for asthma using GWAS data from the UK Biobank (14), plasma pQTL data from Zheng’s study (15), and brain pQTL data from Robins’ study (16). Next, key results were then validated by Bayesian co-localization analysis, reverse causality testing, and phenotype scanning. Additionally, we created a map of the interaction network between the identified proteins, both within the plasma and brain, and with the targets of current asthma medications. We also performed a Kyoto Encyclopedia of Genes and Genomes (KEGG) pathway enrichment to the analysis of the identified proteins and current asthma medication targets. To further strengthen our conclusion, we replicated the analysis using GWAS data from the research by Demenais et al. (17) and the FinnGen cohorts (18) as external validation. **Figure 1** illustrates the design of the study.

## 2 Materials and methods

### 2.1 Data sources and pQTL selection

#### 2.1.1 Plasma pQTL selection

The plasma pQTL data were obtained from a study conducted by Zheng et al. (15). Only pQTLs that met the following criteria were considered: i) exhibited genome-wide significant association (*P* < 5 × 10^−8^), ii) resided beyond the chr6 major histocompatibility complex (MHC) region, iii) showed independent association (LD clumping *r* ^2^ < 0.001), and iv) were *cis*-pQTLs.

#### 2.1.2 Brain pQTL selection

Brain pQTL data are from a study by Robins et al. (16) that reported 786,632 pQTLs for 7,376 brain proteins. These data have been checked for the plausibility of the original file. To address any missing information in the QTL GWAS summary statistics, such as effect allele frequency (eaf), we utilized the corresponding human genome structure as a reference to fill in the materials.

#### 2.1.3 GWAS summary statistics of asthma

Summary statistics from the UK Biobank were used for the primary analysis (14), including 462,933 individuals (until 2017, nCase = 53,598, nControl = 409,335) of European ancestry. Summary statistics from a study by Demenais et al. (17) (nCase = 19,954, nControl = 107,715) and the FinnGen cohorts (R9 release, nCase = 42,163, nControl = 202,399) (18) were used for the external validation.

### 2.2 MR analysis and external validation

In this study, we performed MR using the R programming language (version 4.2.1) with plasma and brain proteins as exposure points and asthma as the outcome, with the TwoSampleMR R package (Version 0.5.6) (19). Wald ratio was used if only one pQTL was available for a particular protein. Inverse variance weighted MR (MR-IVW) was applied when two or more genetic instruments were applicable, and then heterogeneity analysis was performed (20). Odds ratios (ORs) for increased asthma risk were expressed as the standard deviation (SD) of the increase in plasma protein levels and each 10-fold increase in brain protein levels.

The primary analyses were adjusted for multiple tests using Bonferroni correction (21). MR was externally validated only for tentatively identified proteins with a *P*-value threshold set at 0.05.

### 2.3 Sensitivity analysis

#### 2.3.1 Bayesian co-localization analysis

We use the “coloc” R package (Version 5.1.0) (22) with predefined parameters and apply Bayesian collocation analysis to approximate the likelihood of two characteristics having a common causative variable. As mentioned earlier (23), Bayesian co-localization provides four hypothetical posterior probabilities of whether a single mutation is shared by two traits. The study aimed to test two hypotheses—hypothesis 3 (PPH3) and hypothesis 4 (PPH4)—that suggest a relationship between protein and asthma through different or shared variants in a specific region. The criterion used to determine the co-localization of genes was a gene-based PPH4 score of over 80% (24).

#### 2.3.2 Phenotype scanning

We conducted phenotype scanning to search for associations between the identified pQTLs and other traits through the “PhenoScanner” R package (Version 2) (25). A single nucleotide polymorphism (SNP) was deemed pleiotropic if it met the following criteria: i) the association was genome-wide significant (*P* < 5 × 10^−8^); ii) it was only observed in populations of European ancestry; and (iii) the SNPs were associated with any known asthma risk factors, including clinical or metabolic features, proteins, or biological processes. Additionally, we calculated LD *r* ^2^ between pQTLs of preferred proteins to identify possible linkages.

#### 2.3.3 Reverse causality analysis

We performed bidirectional two-sample univariable MR (26) and Steiger filtering to detect the reverse causality of our primary MR analysis. We estimated the effect using MR-IVW, MR-Egger, weighted median, simple mode, and weighted mode and performed a Steiger filtering using a significance threshold of *P <*0.05 to determine the directionality of the protein–asthma association, as a supplement.

### 2.4 Comparison analysis and the analysis with current asthma drug targets

#### 2.4.1 Comparison of the analyzed proteins in the plasma and brain

To investigate the correlation between common brain and plasma PQTLs, effect estimation was performed using MR analysis and analyzed using Spearman correlation analysis. The study also explored the effect of different *P*-value thresholds on the correlation, to determine if significance level had an impact.

#### 2.4.2 Protein–protein interaction network analysis

This study investigates the protein**–**protein interaction (PPI) network of proteins that are potentially associated with asthma risk in the brain or plasma analysis. The objective is to investigate the correlations between the selected proteins and whether the proteins detected through plasma data have any interaction with the ones identified through brain data. The study also investigates the connections between proteins linked to asthma and the targets of drugs that are already available in the market. To achieve this, 19 disease-modifying drugs for asthma were obtained from recent reviews (7, 8) and corresponding drug targets based on the DrugBank database (https://www.drugbank.ca) (27). Additionally, the study searched for existing medications targeting the identified potential causative proteins and interacting proteins by PPI analysis. All PPI analyses were performed using the Search Tool for the Retrieval of Interacting Genes (STRING) database (version 11.5) (https://string-db.org/). A minimum interaction score of 0.4 was set as the requirement for inclusion in the analysis (28).

#### 2.4.3 KEGG pathway enrichment analysis

We performed KEGG enrichment analysis (29) on the genes of current asthma drug targets and potential pathogenic proteins of asthma. Our aim was to investigate the relationship between them and identify new drug targets. This approach will help us better understand the role of these targets in asthma and help us develop new drugs.

## 3 Results

### 3.1 Plasma and brain pQTL selection

The study included 738 *cis*-acting SNPs for 734 proteins, and a total of 73 cis-pQTLs involving 73 proteins were identified based on the aforementioned screening criteria for the plasma pQTL dataset (**Supplementary Table 1**).

### 3.2 Screening the proteome for asthma causal proteins

**Figure 2:**
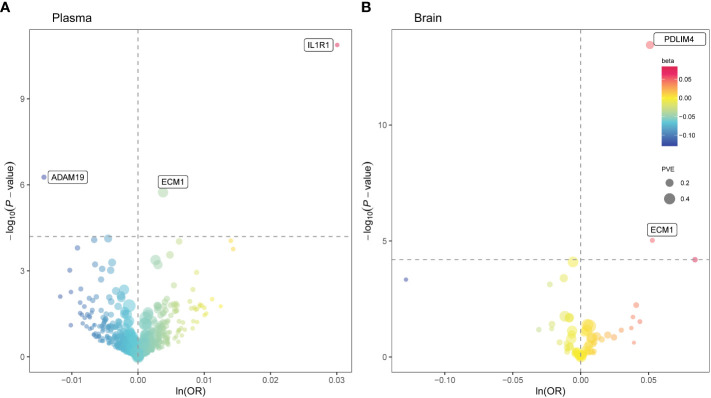
Mendelian randomization (MR) results for plasma and brain proteins and the risk of asthma. Volcano plots of the MR results for **(A)** 734 plasma and **(B)** 73 brain proteins on the risk of asthma. **(A, B)** MR analysis with Wald ratio or inverse variance weighted method on plasma and brain proteins on the risk of asthma, respectively. The ORs for increased risk of asthma were expressed as per SD increase in plasma protein levels and per 10-fold increase in brain protein levels. The dashed horizontal black line corresponded to *P* = 6.35 × 10^−5^ (0.05/788). Ln, natural logarithm; PVE, proportion of variance explained.

At Bonferroni significance (*P* < 6.35 × 10^−5^), MR analysis revealed five protein–asthma pairs (**Table 1**, **Figure 2**), namely, interleukin 1 receptor type 1 (IL1R1), extracellular matrix protein 1 (ECM1), and a disintegrin and metalloprotease domain19 (ADAM19) in the plasma and PDZ and LIM domain 4 (PDLIM4) and ECM1 in the brain. Specifically, increased ADAM19 (OR = 0.99; 95% CI, 0.98–0.99; *P* = 5.40 × 10^−7^) decreased the risk of asthma, whereas elevated IL1R1 (OR = 1.03; 95% CI, 1.02–1.04; *P* = 1.32 × 10^−11^), ECM1 in plasma (OR = 1.00; 95% CI, 1.00–1.01; *P* = 1.83 × 10^−6^), ECM1 in the brain (OR = 1.05; 95% CI, 1.03–1.08; *P* = 9.37 × 10^−6^), and PDLIM4 (OR = 1.05; 95% CI, 1.04–1.07; *P* = 3.55 × 10^−14^) increased the risk of asthma. Preliminary analysis detects no heterogeneity in asthma causal proteins (**Supplementary Table 2**).

**Table 1:** MR results for plasma and brain proteins markedly linked to asthma after Bonferroni-adjusted.

Tissue	Protein	UniProt ID	SNP^a^	Effect allele	OR (95% CI)^b^	*P*-value	PVE	*F* statistics	Author
Plasma	IL1R1	P14778	rs7588201	A	1.03 (1.02, 1.04)	1.32 × 10^−11^	1.21%	39.30	Emilsson
Plasma	ECM1	Q16610	rs13294	A	1.00 (1.00, 1.01)	1.83 × 10^−6^	36.97%	584.28	Suhre
Plasma	ADAM19	Q9H013	rs7728609	C	0.99 (0.98, 0.99)	5.40 × 10^−7^	2.72%	89.52	Emilsson
Brain	PDLIM4	P50479	rs3900945	C	1.05 (1.04, 1.07)	3.55 × 10^−14^	20.86%	16.85	Robins
Brain	ECM1	Q16610	rs698916	G	1.05 (1.03, 1.08)	9.37 × 10^−6^	10.22%	41.00	Robins

a All SNPs used were cis-acting.

b As the levels of plasma protein increased by one standard deviation or the levels of brain protein increased by 10-fold, the risk of asthma also increased.

### 3.3 Sensitivity analysis for asthma causal proteins

**Figure 3:**
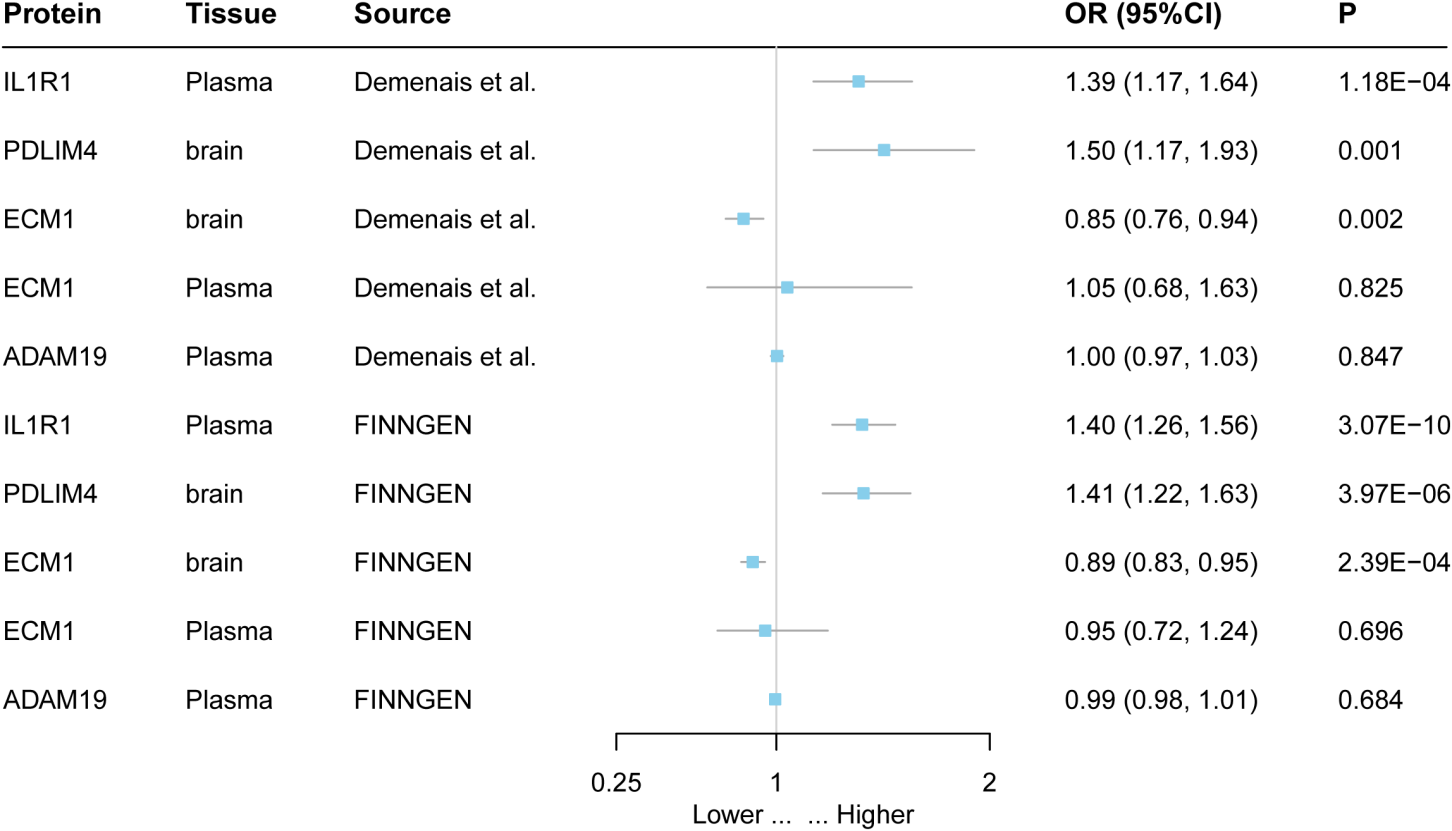
External validation of the causal relationship between five potential causal proteins and asthma. The ORs for increased risk of asthma were expressed as per SD increase in plasma protein levels and per 10-fold increase in brain protein levels.

After conducting sensitivity analysis and MR analysis, we were able to identify three out of the five proteins as potential drug targets for asthma, namely IL1R1, ECM1, and PDLIM4. First, we performed a reverse MR analysis of 102 asthma genetic instruments initially identified by analysis of GWAS data from the UK Biobank (**Supplementary Table 3**) and comprehensive summary statistics for plasma proteins obtained from two previous studies (30, 31). The result reveals no causal effect of asthma on plasma concentrations of the identified proteins (**Figure 3**). Due to a lack of consistent instrumental variables in brain qPTL-GWAS, reverse MR analysis was not performed. Nonetheless, the study utilized Steiger filtering to demonstrate that the proteins identified by the primary MR analysis did not exhibit reverse causality in either plasma or brain, thus providing valuable complementary information to the study’s limitations (**Table 2**, **Supplementary Figure 1**). Second, Bayesian co-localization firmly advised that ECM1 in plasma (coloc.abf-PPH4 = 0.965) and ECM1 in the brain (coloc.abf-PPH4 = 0.931) shared the same variant with asthma (**Table 2**, **Supplementary Figure 2**). Third, phenotype scanning suggested that IL1R1 (rs7588201) is associated with allergic rhinitis (AR), eczema, and allergic disease; ECM1 in plasma (rs13294) is associated with atopic dermatitis. ADAM19 (rs7728609) was found to be associated with pulmonary function. PDLIM4 (rs3900945) is associated with height, carnitine, eosinophil count, blood cell count, and C1q and TNF-related 5 (C1QTNF5). ECM1 in the brain (rs698916) is associated with chronotype (**Table 2**, **Supplementary Table 4**).

**Table 2:** Sensitivity analysis on five potential causal proteins.

Tissue	Protein	UniProt ID	SNP	Bidirectional MR (MR-IVW)^a^	Steiger filtering	Co-localization PPH4 (coloc.abf)	Previously reported
Plasma	IL1R1	P14778	rs7588201	1.09 (0.97, 1.13)	True (1.398 × 10^−8^)	0.002	AR or eczema^b^, allergic disease^c^
Plasma	ECM1	Q16610	rs13294	1.12 (0.91, 1.21)	True (1.989 × 10^−107^)	0.965	Atopic dermatitis^b^
Plasma	ADAM19	Q9H013	rs7728609	1.07 (0.95, 1.16)	True (2.989 × 10^−19^)	0.051	Pulmonary function^c^
Brain	PDLIM4	P50479	rs3900945	NA	True (4.818 × 10^−18^)	0.000	Height^c^, carnitine^b^, eosinophil count^c^; blood cell count^c^, C1QTNF5^b^
Brain	ECM1	Q16610	rs698916	NA	True (9.745 × 10^−9^)	0.931	Chronotype^c^

AR, allergic rhinitis; NA, not available.

a As the levels of plasma protein increased by one standard deviation or the levels of brain protein increased by 10-fold, the risk of asthma also increased.

b SNP directly linked to phenotypic traits.

c SNP with traits that are indirectly influenced through its proxy.

### 3.4 Comparison and PPI analysis of causal proteins in the brain and plasma

A Spearman correlation coefficient of 0.420 was found between the brain and plasma MR results at the protein level. In the analysis, 59 proteins without a *P*-value threshold were included. When the number of proteins was restricted by applying various *P*-value thresholds, a positive correlation still persisted (**Supplementary Figure 3**).

Based on the positive correlation results obtained from the comparison analysis, we proceeded to conduct a PPI analysis of the causal proteins found in both plasma and brain. The results showed that IL1R1 appears to be connected with IL7R, intercellular adhesion molecule 1 (ICAM1), and IL6ST via text mining and co-expression, and also IL1R1 might be connected with IL1R2 via protein homology, gene co-occurrence, curated databases, and test mining. In addition, ADAM19 might be connected with cadherin 11 (CDH11) and nephronectin (NPNT) (**Supplementary Figure 4**).

### 3.5 Relationship between potential drug targets and existing asthma drugs and targeted drugs

**Figure 4:**
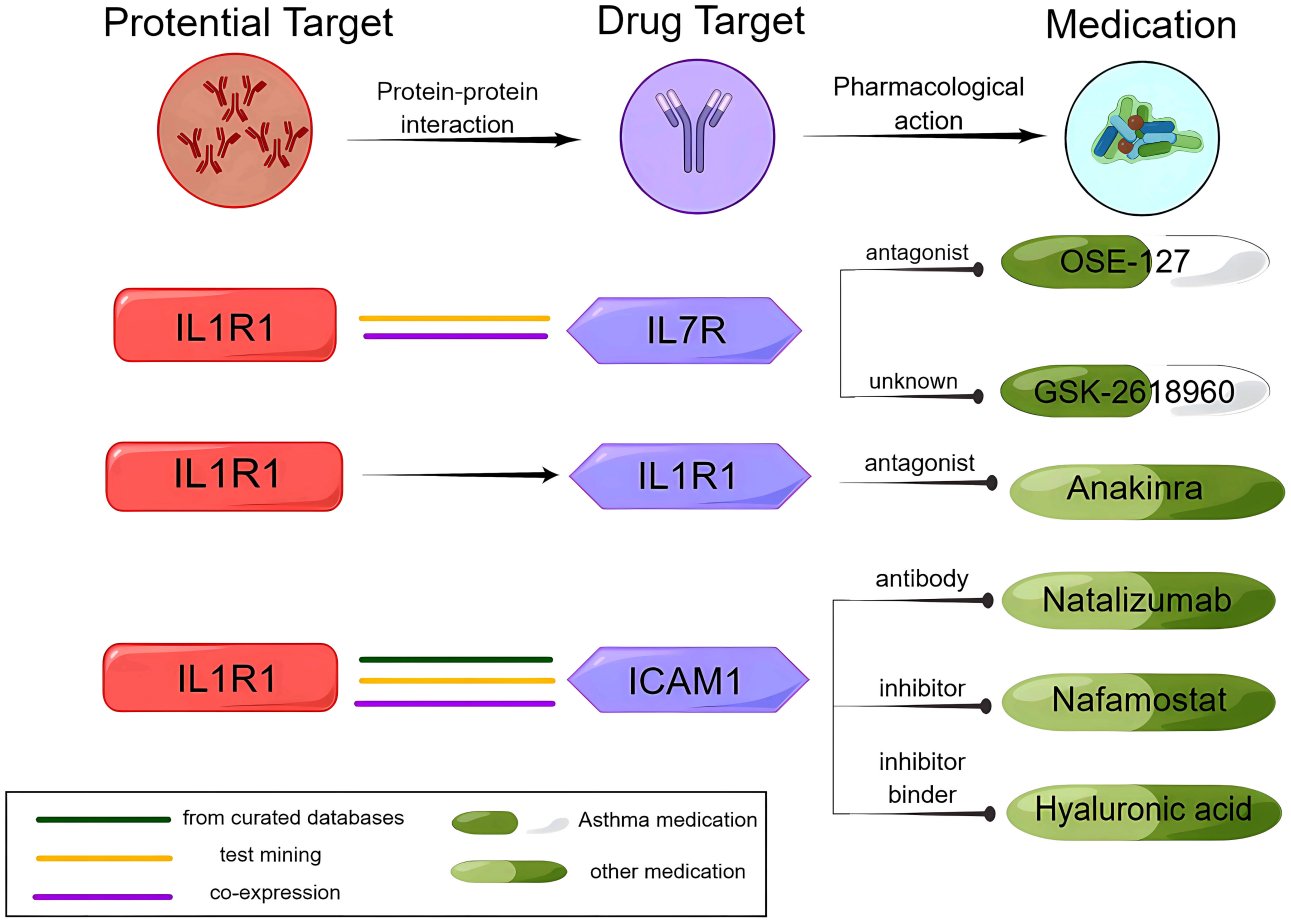
Analysis of the potential drug target IL1R1, the IL1R1 interaction protein ICAM1, and medications.

IL1R1 and IL7R were determined to have strong reliable interactions (known interactions experimentally determined) by STRING. IL1R1 was associated with anti-IL7R monoclonal antibodies (IL7R), the target of OSE-127 and GSK-2618960. STRING also revealed that IL1R1 and ICAM1 have the most reliable interactions (from curated databases). Natalizumab, nafamostat, and hyaluronic acid are medications that target ICAM1. Under the framework of drug repositioning, investigating the therapeutic potential of these drugs for asthmatic patients is a valuable pursuit (**Figure 4**, **Supplementary Figure 4**, **Supplementary Tables 5, 6**).

We conducted a search in the DrugBank database for current medications that target the proteins identified as potential causal factors. One medication that might modify the disease was identified: anakinra (an antagonist of IL1R1) (**Supplementary Table 6**).

Moreover, the KEGG analysis revealed the enrichment of genes for the causal protein IL1R and known asthma drug target genes in pathways related to infection, inflammation, and immune cell differentiation (**Supplementary Table 7**).

### 3.6 External validation of potential drug targets for asthma

The findings of this study were replicated in different datasets, indicating that IL1R and PDLIM4 are associated with asthma. Both the FinnGen cohort and the GWAS data from a study by Demenais et al. showed that increasing IL1R1 increased the risk of asthma. For example, increasing PDLIM4 increased the risk of asthma (OR = 1.41; 95% CI, 1.22–1.63; *P* = 2.39 × 10^−6^) in the FinnGen cohort (**Figure 3**, **Supplementary Table 8**).

## 4 Discussion

This study is the first to employ a two-sample MR approach by combining plasma and brain proteomic data to investigate causal proteins for asthma. The study identified three potential drug targets for asthma, including IL1R1, ECM1, and PDLIM4. Bayesian co-localization analysis suggested that ECM1 likely shares the same variant of asthma. Additionally, IL1R1 and PDLIM4 were found to be associated with asthma in two other large asthma GWAS datasets, further supporting the reliability of the potential drug targets identified in this study.

MR analysis uses genetic variation as instrumental variables (IVs); IVs affected the exposure but not the outcome. Furthermore, IVs only had an impact on the outcome when they were involved in the exposure pathway and not through any other means.

Hence, we applied a series of assessments and sensitivity analyses to interpret our results in the primary MR analysis. i) Weak instrument bias: We calculated the *F* statistics (32) and the proportion of variance explained (PVE) (33) of the SNPs in our investigation, and the SNPs for the top findings showed that all the SNPs were strong instruments with *F* statistics >10, and most SNPs had PVE more than 10%, except for plasma IL1R1 and ADAM19. ii) Heterogeneity of the SNPs: Cochran’s *Q* test did not reveal any significant heterogeneity in plasma pQTL. In addition, since only one SNP is included for each protein in brain tissue, it is not possible to perform heterogeneity tests. iii) Horizontal pleiotropy: Considering the direct role of SNPs in the transcription and/or translation of the associated genes, we only utilized *cis*-pQTLs screening all of the genomes for IVs with a significance threshold less than 5 × 10^−8^ (34). Also, we found that the protein targets were linked to various traits through phenotype scanning. However, none of these associations were sufficient to fully elucidate the relationship between the identified proteins and asthma. The results showed the associations between SNP and multiple allergic diseases and the co-occurrence of asthma and allergic rhinitis (35), eczema (36), and atopic dermatitis (37). This suggests that the aforementioned diseases may share a common etiology. PhenoScanner also revealed that C1QTNF5 is associated with rs3900945 but in a *trans*-acting manner, rendering them less likely to bias the PDLIM4–asthma association. However, carnitine is associated with PDLIM4. Studies conducted earlier have demonstrated a reduction in serum carnitine levels during asthma exacerbation (38) and also in severe asthma with oral corticosteroids (OCS)-independent (39). Therefore, the possibility that carnitine affects the relationship between PDLIM4 and asthma cannot be completely ruled out, and the role of PDLIM4 needs to be interpreted cautiously. iv) Genetic confounding due to LD: Only summary statistics from GWAS conducted on individuals of European ancestry were utilized for MR to avoid genetic confounding caused by LD. Moreover, Bayesian co-localization was also utilized. v) Reverse causality: Steiger filtering revealed that none of the proteins identified in the primary MR analysis exhibited reverse causality (40). This finding was further validated by bidirectional MR analysis.

Asthma is a heterogeneous disease, and the current treatments focus on alleviating symptoms (41). However, targeted therapy based on etiology and pathological mechanisms can ultimately lead to better outcomes for asthma patients. While numerous novel targets for asthma have been identified in recent years, using these discoveries to exploit new drugs or repurpose existing drugs to treat asthma remains challenging. Zaid et al. (7) discovered a series of asthma drug targets based on GWAS and eQTL analyses, whereas our study applied pQTL. It is important to validate a credible link between an asthma SNP and gene expression by identifying relevant cell types that express the gene and validating mRNA expression at the protein level. So, our study adds to the existing research by Zaid et al. on drug targets of asthma, which are crucial for the success of market approval.

In addition, the significance of neurogenic inflammation in asthma development led us to select brain pQTL as one of the exposures for MR analysis. In the lungs, sensory nerves detect chemicals and stimuli and cause bronchoconstriction by activating central neural pathways (42). In asthmatics, eosinophil-released mediators increase sensory nerve density in the lung epithelium, resulting in hyperactivity to environmental stimuli (43). Although the evidence is still in its initial stages, the findings imply that protein targets associated with asthma can be identified through both the brain and plasma.

ECM1 appears to be the only potential target with activity in both the brain and plasma. ECM1 is a soluble glycoprotein with a molecular weight of 85 kDa (44). Its primary functions include stimulating endothelial cell proliferation and inducing angiogenesis (45). Additionally, it combines with collagen IV to stimulate the activity of collagenase, which is relevant to patients with autoimmune diseases (46, 47). As previous research on ECM1 has reported conflicting directions of effects, the relationship between ECM1 levels and asthma remains uncertain. Zhenhu Li et al. demonstrate that ECM1 is elevated and specifically expressed in Th2 cells, leading to exacerbated allergic airway inflammation *in vivo* (48). In contrast, another study found that ECM1 inhibits the differentiation of Th17 cells in central nervous system inflammatory diseases, but the inhibition of Th17 cell differentiation can reduce the occurrence of asthma (49). Our study identifies ECM1 as a risk factor for asthma. The viewpoints presented indicate that additional research is necessary to investigate the direction of the relationship between ECM1 and asthma. Although tissue-specific expression patterns of ECM1 may account for the observed discrepancy, the fact that it is the only protein co-localized in both brain and plasma indicates a higher probability of it being a potential therapeutic target.

Previous studies have found that PDLIM4 promotes the synaptic accumulation of the α-amino-3-hydroxy-5-methyl-4-isoxazolepropionic acid (AMPA) receptor, in an alpha-actinin/actin-dependent manner, in the central nervous system (CNS) (50). The modulation of respiratory rhythm is affected by the activity of AMPA receptors. When these receptors are activated, it results in an increase in rhythmic neuron depolarization and respiratory rhythm frequency, thereby exacerbating asthma (51). Although our PPI analysis did not reveal any interactions between PDLIM4 and current asthma medications, our study validated PDLIM4 as a potential drug target for asthma through external validation in two separate cohorts. Therefore, we posit that PDLIM4 may hold promise as a therapeutic target for treating asthma.

IL1R1 is a receptor that, when bound to IL-1α and IL-1β, activates intracellular signaling pathways. A previous study showed that glucose metabolism in the amygdala increased during psychological stress in patients with asthma, which was associated with increased IL-1 signaling in the airways, suggesting the existence of brain–immune pathways in asthma (52). In our study, IL1R1 interacted with IL7R in the PPI analysis. IL7R is expressed most abundantly in T cells (53). According to the druggable genome research screening (54), IL7R, which encodes a receptor protein, is considered a gene with the potential as a target for small molecule therapeutic drugs. IL7R, when combined with IL7 or TSLP, can activate the JAK–STAT and other pathways and plays a regulatory role in type 2 inflammation (55, 56). Preclinical studies have indicated that OSE-127, a drug targeting IL7R, is effective for asthma (7). Furthermore, teciprumab, a targeted drug corresponding to TSLP, is currently undergoing a phase 3 clinical trial in patients with poorly controlled asthma (57). Moreover, our analysis of PPI revealed that IL1R1 had an interaction with ICAM1, the treatment target for natalizumab, nafamostat, and hyaluronic acid. As the concept of drug repositioning has been applied to drugs that are currently on the market or under development (58), this approach can be used to investigate whether the three aforementioned drugs can also be effective in treating asthma. Since the safety of these drugs has already been established, this method can improve the efficiency of drug development while reducing costs and time. Moreover, IL1R is associated with known asthma drug target genes in KEGG pathways related to infection, inflammation, and immune cell differentiation. Together, IL1R1 might be a potential therapeutic target for asthma.

There are some limitations in our study. First, while this study examined patients with various types of asthma, it is possible to identify variants for specific subsets of patients, such as those with childhood-onset asthma or frequent episodes of exacerbations, or patients with specific endotypes. Future research should aim to include more clinical information and incorporate a greater amount of pQTL data from specific inflammatory cell types. This will enable a more personalized approach to asthma treatment. Future research should incorporate additional clinical information to facilitate a more personalized approach to asthma treatment. Second, our analysis was conducted on populations of European ancestry to minimize the bias from population stratification. However, it should be noted that our findings may not be as applicable to other ancestries. While we also performed causal inferences in the Finnish populations, we plan to conduct additional studies once we have more asthma GWAS data on other ancestry. This will allow us to further explore our research findings and facilitate the translation of our findings into clinical applications. Third, strict screening conditions, such as genome-wide significant association (*P* < 5 × 10^−8^) and *cis*-acting pQTL, enable MR analysis without horizontal pleiotropy but also limit the application of some MR analysis methods. However, by calculating the *F* statistic, we found that most of the instrumental variables we screened have an *F* statistic greater than 10. We obtained the effect allele frequency for plasma pQTLs from a compatible human genome reference, and the relevant information concerning ADAM19 demonstrated proximity to 0.5, making its effect direction less reliable. In previous studies, it was found that ADAM19 was mainly expressed in the apical part of the bronchial epithelium (59), which plays a role in the remodeling of airways and vasculature (60). Consequently, the roles of ADAM19 ought to be cautiously construed. Finally, our analysis of protein targets and drug targets of current asthma medications through PPI and KEGG pathway analysis did reveal some potential interactions. It is crucial to highlight that these outcomes are merely suggestive, and additional research is necessary to validate these findings, such as studies in *in-vitro* cell lines, animal models, and clinical samples.

## 5 Conclusion

Our study demonstrates a causal relationship between genetically determined IL1R1, ECM1, and PDLIM4 plasma protein levels and asthma and, additionally, a causal relationship between ECM1 protein levels in brain tissue representing neurogenic inflammation and asthma. This finding suggests that IL1R1 and ECM1 could be promising targets for drug development in the treatment of asthma. However, further research is necessary to gain a complete understanding of the roles of these proteins in the development and progression of asthma.

## Data availability statement

The original contributions presented in the study are included in the article/**Supplementary Material**. Further inquiries can be directed to the corresponding author.

## Ethics statement

This study was not required to obtain approval from the Ethical Review Authority as the data used were already publicly available, anonymized, and de-identified. The studies were conducted in accordance with the local legislation and institutional requirements. The human samples used in this study were acquired or gifted from another research group. Written informed consent for participation was not required from the participants or the participants’ legal guardians/next of kin in accordance with the national legislation and institutional requirements.

## Author contributions

JW designed the study and supervised the project. YW performed all the Mendelian randomization analyses described here. YW, WX, and SL searched the literature. YW, ZY, and SL wrote and edited the manuscript. ZY and SL collected and examined the data. All authors contributed to the article and approved the submitted version.
