# Supplementary material for: Potential drug targets for asthma identified in the plasma and brain through Mendelian randomization analysis
Source: Front Immunol. 2023 Sep 21;14:1240517. doi: 10.3389/fimmu.2023.1240517 (PMC10551444; doi:10.3389/fimmu.2023.1240517)
Supplement: Supplementary file 1 [file DataSheet_1.doc]

Supplementary Material

# Potential drug targets for asthma identified in plasma and brain through Mendelian randomization analysis

**Yuting Wang^1^, Jiaxi Wang^1^*, Zhanfeng Yan^2^, Siming Liu^2^ and Wenlong Xu^2^**

*** Correspondence:** Jiaxi Wang: b00704@bucm.edu.cn

# Supplementary Figures and Tables

## Supplementary Figures


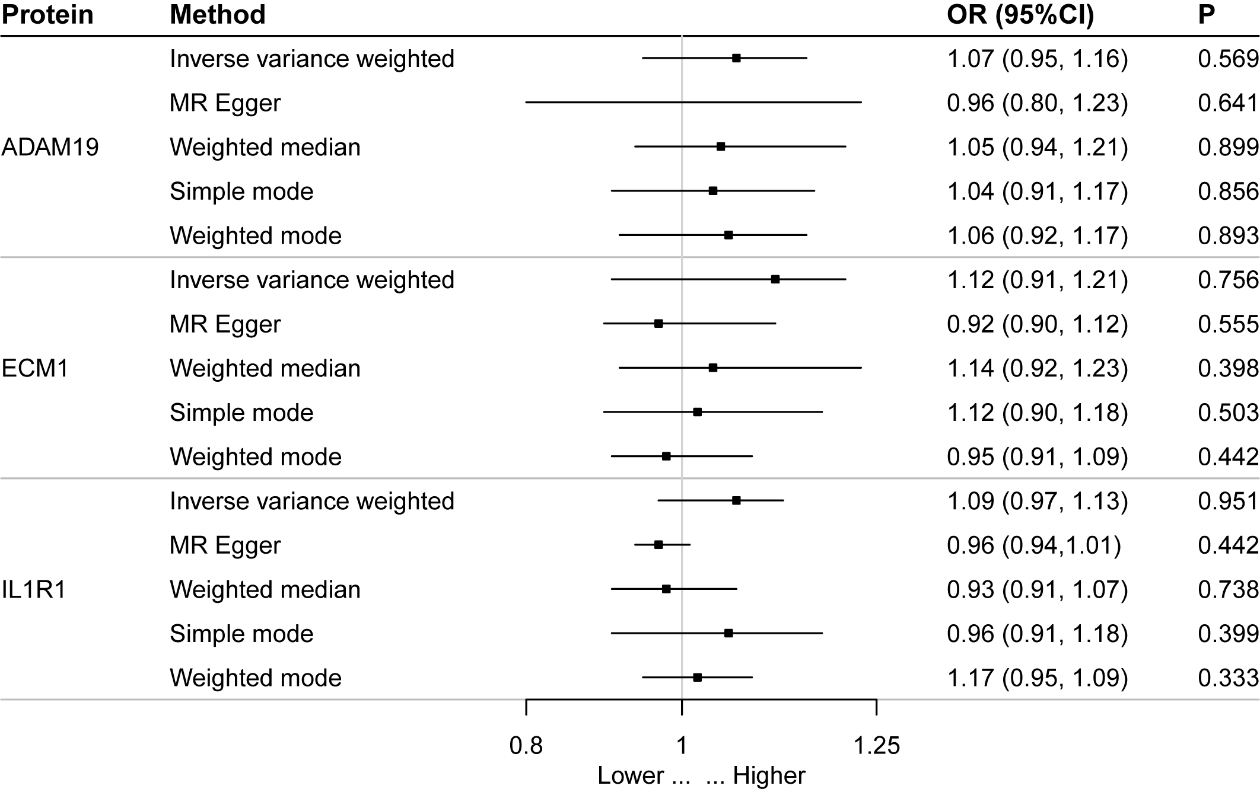


**Supplementary Fig. 1 Bidirectional MR analysis for asthma on levels of three potential causal proteins in plasma**

OR stood for the odds ratios for per standard deviation (SD) increase in plasma protein levels as asthma risk increased.


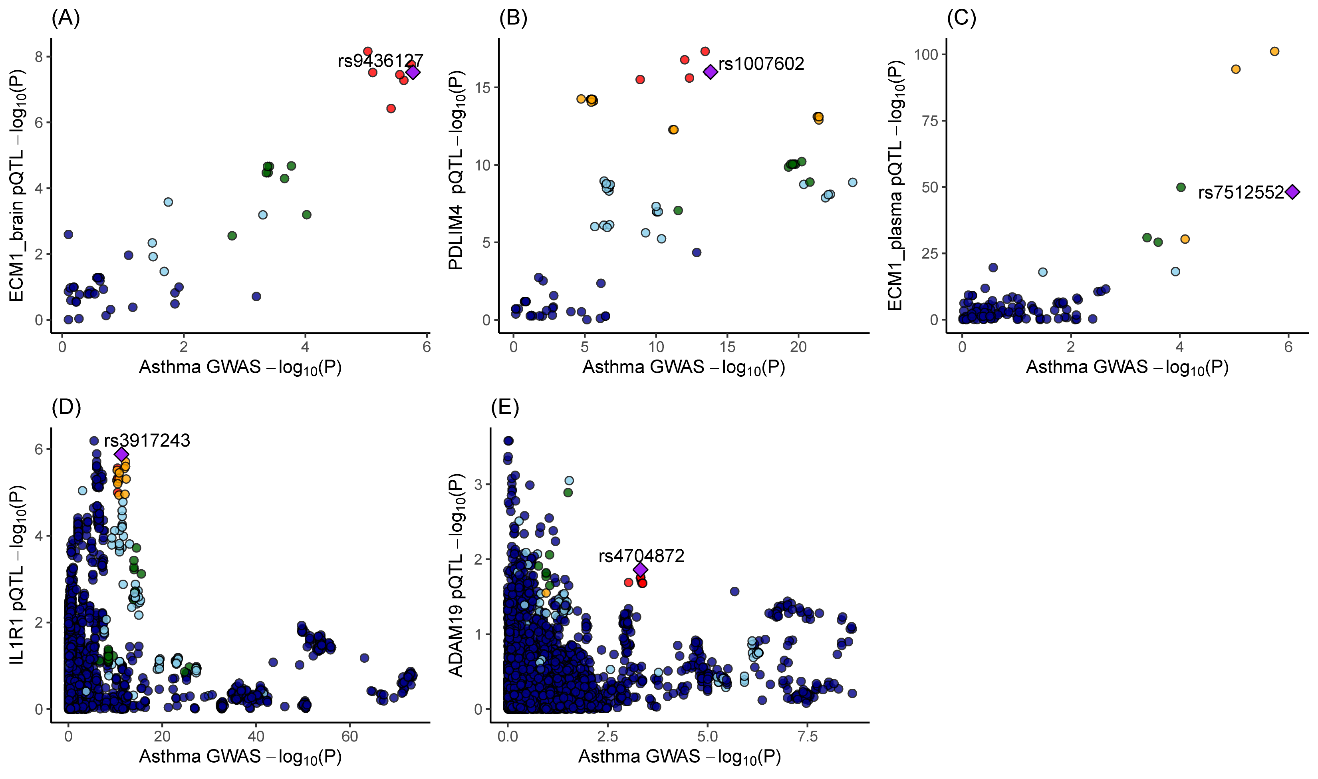


**Supplementary Fig. 2 Bayesian colocalization analysis of five potential causal proteins and asthma**

Colocalization analysis of brain proteins for ECM1 **(A)** and PDLIM4 **(B)**, and plasma proteins for ECM1 **(C)**, IL1R1 **(D)**, and ADAM19 **(E)**, respectively. Diamond purple points represented the SNP with the minimal sum of *P* value in corresponding protein GWAS and asthma GWAS.


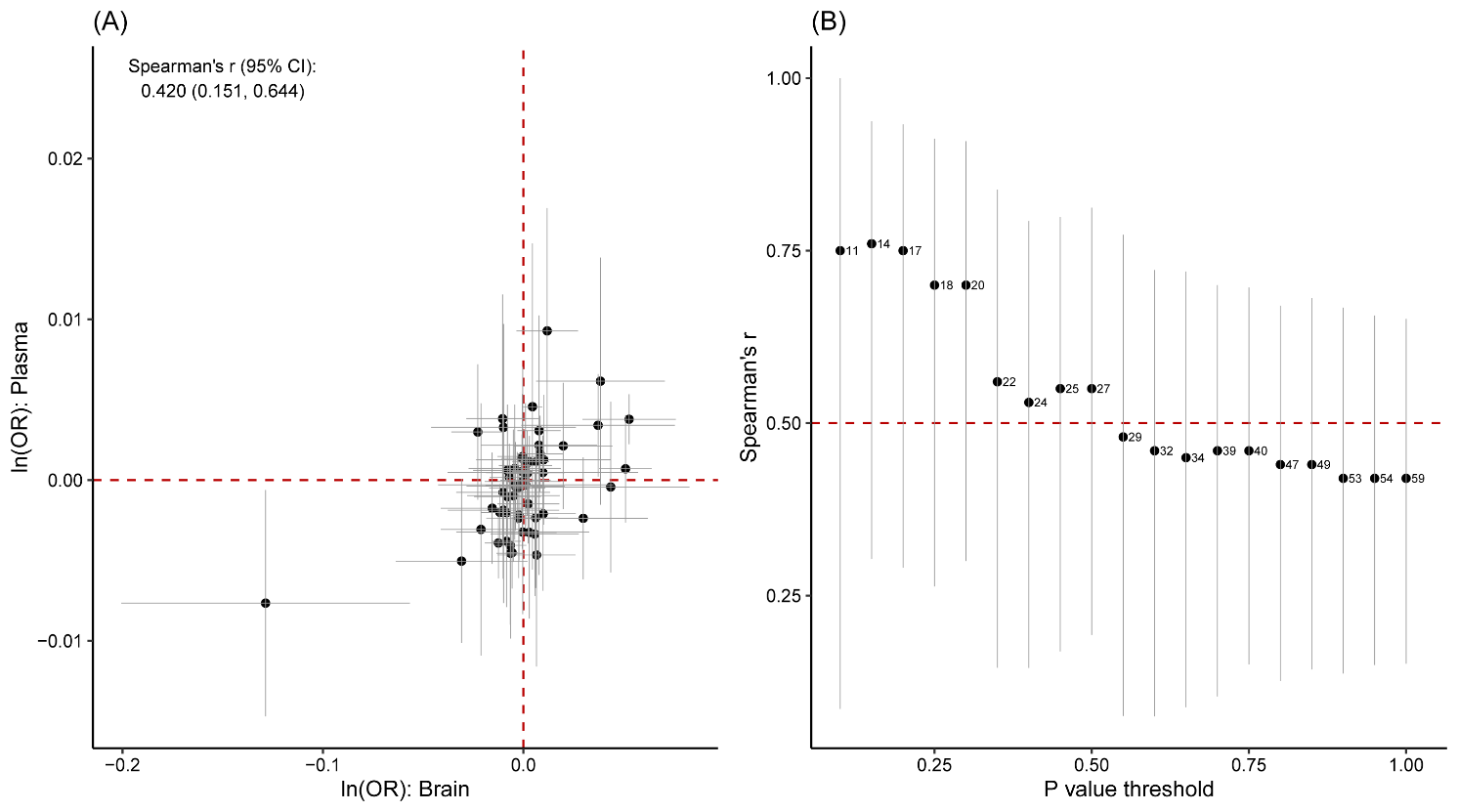


**Supplementary Fig. 3 Comparison analysis of MR estimates between plasma proteome and brain proteome**

**(A)** All 59 overlapping proteins in plasma and brain were used to perform correlation analysis. The horizontal and vertical gray lines represented the 95% confidence interval of MR estimates in the main analysis. The Spearman correlation coefficient was 0.420 (95% CI: 0.151, 0.644); **(B)** With different cutoffs for *P* value to include MR estimates, Spearman correlation coefficient was calculated. The numbers on the left side of the black point represented the numbers of overlapping proteins correspondingly.


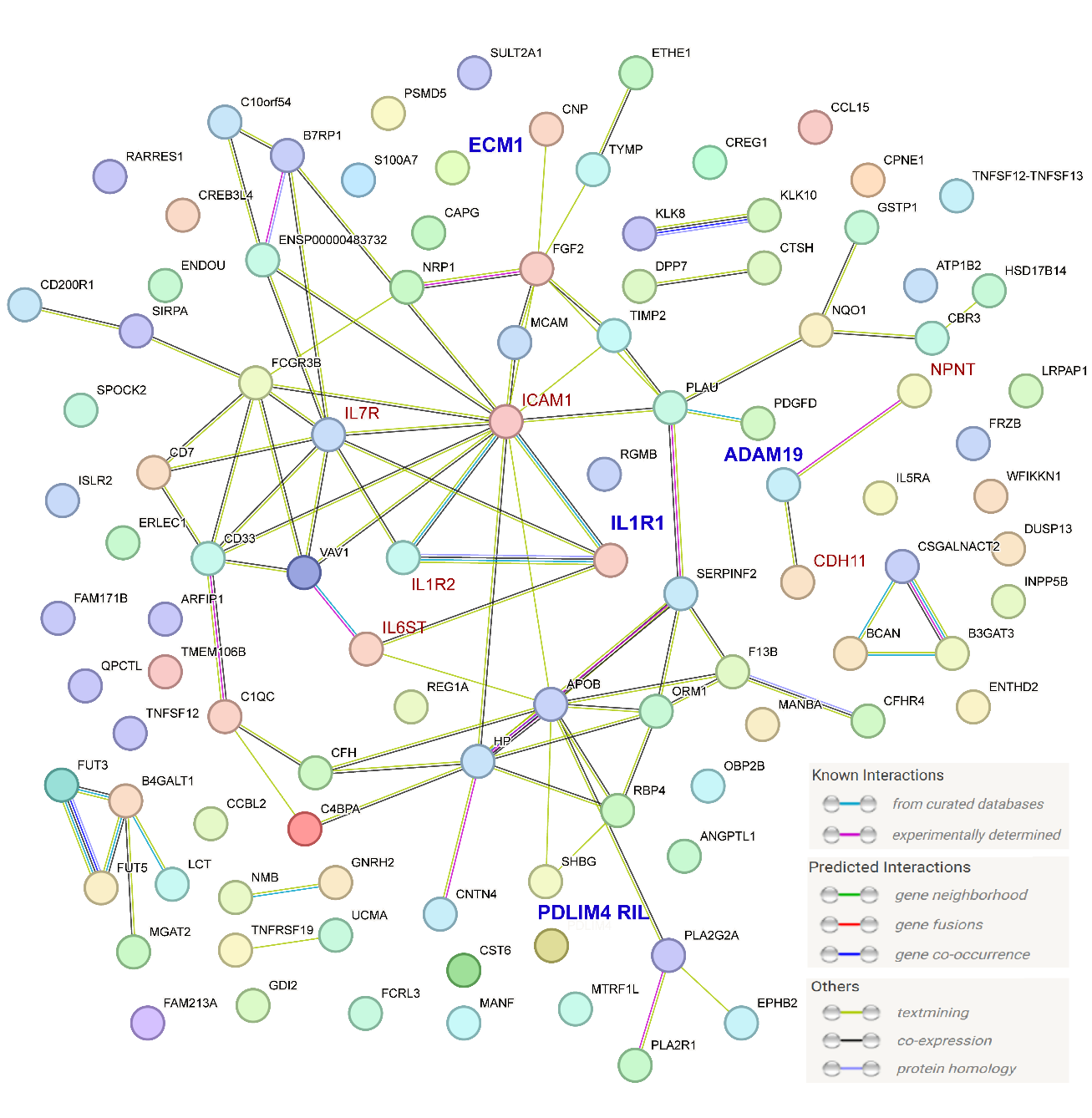


**Supplementary Fig. 4 Protein-protein interaction network among the suggestive causal proteins (*P* < 0.05)**

The black labels represented the suggestive causal proteins (P < 0.05) in plasma and brain. Blue labels represented the five top causal plasma and brain proteins, and Blue labels represented the interacted proteins for current medications.

**1.2 Supplementary Tables**

**Supplementary Table 1** Genetic instruments of plasma and brain proteins for MR analysis

**Supplementary Table 2** Genetic instruments of asthma for bidirectional MR

**Supplementary Table 3** Heterogeneity analysis on proteins with two or more instruments

**Supplementary Table 4** Previously-reported genome-wide significant association of SNPs as genetic instruments of five potential causal proteins

**Supplementary Table 5** Nineteen medications for multiple sclerosis and their corresponding drug targets

**Supplementary Table 6** Current medications targeting five potential causal proteins and interacted proteins

**Supplementary Table 7** KEGG pathway enrichment analysis for five potential proteins and the corresponding drug targets for asthma

**Supplementary Table 8** Genetic instruments of five potential causal proteins for external validation
